# 

**Published:** 1947

**Authors:** 


### Bristol Medico-Chirurgical Society

### The Medical Library of the University of Bristol

WITH WHICH IS INCORPORATED

**The Library of the Bristol Medico-Chirurgical Society.**

*The following donations have been received since the publication of the list in the last issue.*

| | |
|---|---|
| Mrs. F. M. Adams (1) .. .. .. | 56 volumes and pamphlets on radiology. |
| H. Chitty, Esq. (2) .. .. .. .. .. | 1 volume. |
| General Medical Council (3) .. .. .. .. | 5 volumes. |
| Dr. H. S. Heller (4) .. .. .. .. .. | 3 volumes. |
| Mayo Clinic (5) .. .. .. .. .. .. | 4 volumes. |
| Dr. T. Milling (6) .. .. .. .. .. .. | 80 volumes. |
| Wallace and Tiernan Ltd. (7) .. .. .. .. | 1 volume. |
| Professor R. Milnes Walker (8) .. .. .. | 10 volumes. |

Unbound periodicals have also been received from Dr. C. D. Evans, Dr. T. Milling, Professor C. Bruce Perry, Professor R. Milnes Walker, and Messrs. John Wright and Sons, Ltd.

### THE ONE HUNDRED AND EIGHTY-FOURTH LIST OF BOOKS.

The figures in round brackets refer to the figures after the names of the donors. The books to which no such figures have been attached have either been bought from the Library Fund or received through the Journal.

| | | | |
|---|---|---|---|
| **Adriani, J.** | *Chemistry of Anaesthesia* | | 1946 |
| **Barcroft, Sir J.** | *Researches on Pre-natal Life* | Vol. 1 | 1946 |
| **Belding, D. L.** | *Textbook of Clinical Parasitology* | | 1942 |
| **Browne, O'D. T. D.** | *The Rotunda Hospital*, 1745-1945 | | 1947 |
| **Comrie, J. D.** | *Black's Medical Dictionary* | 18th Ed. | 1946 |
| **Conant, N. F. and others** | *Manual of Clinical Mycology* | | 1946 |
| **Dilling, W. J. and Hallam, S.** | *Dental Materia Medica, Pharmacology and Therapeutics* | 3rd Ed. | 1946 |
| **Dodson, A. I.** | *Synopsis of Genito-urinary Diseases* | 4th Ed. | 1945 |
| **Dunlap, F. L.** | *White versus Brown Flour* | (7) | 1945 |
| **Fairbrother, R. W.** | *Textbook of Bacteriology* | 5th Ed. | 1946 |
| **Frazer, J. E.** | *Anatomy of the Human Skeleton* | 4th Ed. | 1946 |
| **Garrod, A. E. and others** | *Diseases of Children* | Vol. 1. 4th Ed. | 1947 |
| **General Medical Council** | *Recommendations as to the Medical Curriculum* | | 1947 |
| **Gibbens, J.** | *The Care of Young Babies* | 2nd Ed. | 1946 |
| **Glasser, O. and others** | *Physical Foundations of Radiology* | | 1947 |
| **Hale-White, W.** | *Materia Medica, Pharmacy, Pharmacology and Therapeutics* | 26th Ed. | 1946 |
| **Harrison, G. A.** | *Chemical Methods in Clinical Medicine* | 3rd Ed. | 1947 |
| **Hinshelwood, C. N.** | *Chemical Kinetics of the Bacterial Cell* | | 1946 |
| **Horder, Lord (Ed.)** | *British Encyclopaedia of Medical Practice: Medical Progress* 1947*: Cumulative Supplement* 1947 | | 1947 |
| **International Labour Office** | *The Training and Employment of Disabled Persons* | (2) | 1945 |
| **Johnston, T. B.** | *Synopsis of Regional Anatomy* | 5th Ed. | 1945 |
| **Lawrence, R. D.** | *The Diabetic A.B.C.* | 9th Ed. | 1946 |
| **Lea, D. E.** | *The Action of Radiations on Living Cells* | | 1946 |
| **Lee, J. A.** | *Synopsis of Anaesthesia* | | 1947 |
| **McDowall, R. J. S.** | *Handbook of Physiology and Biochemistry* | 39th Ed. | 1946 |
| **McGregor, A. L.** | *Synopsis of Surgical Anatomy* | 6th Ed. | 1946 |
| **Mach, R. S.** | *Les Troubles du Métabolisme du sel et de l'eau* | | 1946 |
| **Moncrieff, A., and Thomson, W. A. R. (Ed.)** | *Child Health* | | 1947 |
| **Peters, J. P. and Van Slyke, D. D.** | *Quantitative Clinical Chemistry*, Vol. 1. *Interpretations* | 2nd Ed. | 1946 |
| **Pinner, M.** | *Pulmonary Tuberculosis in the Adult* | | 1945 |
| **Purves-Stewart, J.** | *Diagnosis of Nervous Diseases* | 9th Ed. | 1947 |
| **Robertson, E. G.** | *Further Studies in Encephalography* | | 1946 |
| **Rolleston, H. and Moncrieff, A. (Ed.)** | *Modern Anaesthetic Practice* | 2nd Ed. | 1946 |
| **Sequeira, J. H., and others** | *Diseases of the Skin* | 5th Ed. | 1947 |
| **Stallard, H. B.** | *Eye Surgery* | | 1946 |
| **Stevenson, R. S.** | *Morell Mackenzie* | | 1946 |

TRANSACTIONS, REPORTS, JOURNALS, ETC.

Annals of Medical History Index. 1917-1942 .. .. .. .. .. .. .. .. 1946

Collected Papers of the Mayo Clinic. Vols. 5–8 .. .. .. (5) 1913–1916

Dentists' Register, 1947 .. .. .. .. .. .. .. .. .. .. .. .. (3) 1947

Farmacoterapia Actual. Vols. 1–3 .. .. .. .. .. .. .. .. (4) 1944–1946

Journal of Thoracic Surgery. Vols. 6–15 .. .. .. .. .. .. (8) 1937–1946

Medical Register, 1947 .. .. .. .. .. .. .. .. .. .. .. (3) 1947

Minutes of the Dental Board of the United Kingdom. Vols. 22–24 (3) 1943–45

Yearbook of Dermatology and Syphilology, 1945 .. .. .. .. .. .. 1945

Yearbook of General Medicine, 1945 ; 1946 .. .. .. .. .. 1945–1946

Yearbook of General Surgery, 1945 ; 1946 .. .. .. .. .. .. 1945–1946

Yearbook of Neurology, Psychiatry and Endocrinology, 1945 .. .. .. 1945

Yearbook of Obstetrics and Gynecology, 1946 .. .. .. .. .. .. .. 1946

Yearbook of Pediatrics, 1946 .. .. .. .. .. .. .. .. .. .. 1946

Yearbook of Urology, 1945 ; 1946 .. .. .. .. .. .. .. 1945-1946

### Publications Received

From MESSRS. JOHN WRIGHT & SONS, LTD. :

*A synopsis of anaesthesia.* By J. ALFRED LEE, M.R.C.S., L.R.C.P., M.M.S.A., D.A. Price 12s. 6d. 1947.

*Massage and remedial exercises in medical and surgical conditions.* By NOEL M. TIDY. 7th Edition. Price 25s. 1947.

From OXFORD UNIVERSITY PRESS :

*Heparin in the treatment of thrombosis.* By J. ERIK JORPES, M.D. 2nd Edition. Price 18s. 1946.

*Elements of surgery.* By FAUSET WELSH, B.Sc., M.B., F.R.C.S. Price 7s. 6d. 1947.

From MESSRS. H. K. LEWIS & CO., LTD. :

*A textbook on the nursing and diseases of sick children.* Edited by ALAN MONCRIEFF, M.D., F.R.C.P. 4th Edition. Price 30s. 1947.

*Clinical examination of the nervous system.* By G. H. MONRAD-KROHN, M.D., F.R.C.P. 8th Edition. Price 16s. 1947.

From MESSRS. W. HEFFER & SONS, LTD.

*Anatomical terms : their origin and derivation.* By E. J. FIELD, M.D., M.S. and R. J. HARRISON, M.A., M.B., B.Chir. Price 7s. 6d. 1947.

### Local Medical Notes

BRISTOL UNIVERSITY.

The following appointments have been made: *Professor of Child Health*, Albert Victor Neale, M.D., F.R.C.P., D.P.H. *Lecturer in Forensic Medicine.*—C. R. Gibson, M.B., Ch.B. *Lecturer in Children's Dentistry.*—J. H. Glen, L.D.S. *Clinical Teacher in Radiology.*—J. P. Raban, M.R.C.S., L.R.C.P., D.M.R.E. *Clinical Teacher in Fevers.*—James Macrae, M.D., F.R.F.P.S., D.P.H. *Recognised Teacher for Health Visitors Training Course.*—A. L. Smallwood, M.B., Ch.B., D.C.H., D.P.H. *Additional Medical Officer to the University.*—B. W. Davy, M.B., B.S.

EXAMINATION RESULTS.

**University of Bristol.**—Students of the University have recently passed the following examinations:—

M.B., CH.B.—*First Examination:* F. J. Applebe, K. J. Barker, D. F. Brittain, Sheila F. Carey, B. J. F. Coles, P. A. Crook, R. W. Digby, H. G. Gage, E. C. Gawthorn, E. P. Hamblett, G. P. Lowther, Violet E. Marsden, Sheila A. Martyn, M. N. Pritchard, T. D. Richards, N. G. Sanerkin, P. H. Sherwood, H. S. Smith, D. W. G. Stone, J. E. Tovey, H. A. P. Walker. *In Organic Chemistry and Physics (completing the Examination):* Anne W. Christie.

M.B., CH.B.—*Second Examination:* T. E. Dada, M. E. Hincks, G. W. B. Peel, G. A. Pilkington, Mildred M. C. Shaw. *In Section II (completing the Examination):* R. J. Ancill, (Mrs.) Helena B. M. Bailey, P. J. D. Benjafield, Monica B. Bishop, H. J. Bland, K. A. Boulton, Margaret H. Buston, C. E. Dickson, Dorothy R. Douglas, E. R. Duchesne, W. A. W. Dutton, Doreen A. Ellis, Luba Epstejn, B. R. W. Evans, (Mrs.) Hella R. Fluss, Z. Fluss, R. F. Harvey, R. Hill, E. A. W. Houghton, J. A. Jefferies, H. I. Leigh, Jean M. Liell, H. Maxwell, J. Oates, Barbara M. Philpott, J. F. Rowland, Rachel D. R. Sasieni, M. Sheldrick, (Mrs.) Antonina J. Sidorowicz, L. P. Spence, D. T. I. Stuttaford, Marianne A. Taylor, Ginette M. Tebbutt, P. A. Titterton, L. A. D. Tovey, M. D. Turner, Joan Walker. *In Section I only:* S. H. Brown, Megan D. M. Johnson, J. H. Scudamore, Patricia Vowles.

M.B., CH.B.—*Final Examination:* C. A. Mills (with Second Class Honours, Distinction in Medicine and in Public Health), G. F. Bigwood (with Distinction in Obstetrics), F. G. Bolton, W. L. Codd, Margaret J. Cox, W. J. Gall (with Distinction in Surgery), G. J. S. Herdman, Stella C. Hill (with Distinction in Obstetrics), J. A. G. Holt (with Distinction in Public Health), Mary Knowles, Lisa E. Mandelbaum, G. E. Mitchelmore, W. H. N. Moore, G. G. Portch (with Distinction in Surgery and in Public Health), E. Saphier, (Mrs.) Patricia M. Valentine, M. H. Weiss. *In Group II:* Patricia M. Appleton. *In Group I only:* (Mrs.) Elizabeth H. Chard, C. E. Halliday, Kathleen C. Iles. *In Group II only:* Catherine M. Hoyle.

M.B., CH.B.—*Final Examination* (*Section I*) *:* Barbara Brosnan, R. J. Carey, Suzanne K. R. Clarke, Anne B. Cousins, Eudora M. R. Davies, Vera H. M. Dowling, D. N. Fitzgerald, Molly I. Govier, B. T. Hale, Pamela I. Hannaford, Kathleen J. Harrison, Catherine M. Hoyle, Mary Knowles, A. J. Lee, A. H. Levy (with Distinction in Materia Medica, Pharmacy, etc., and in Pathology and Bacteriology), C. D. Linton, J. S. R. R. Old, D. B. Peacock, Jenny Pym, F. A. A. Ruggeri, G. T. Salter, A. S. Wallace.

B.D.S.—*First Examination :* I. Apter, B. G. Bennett, K. R. Cowles, Joan M. Flint, R. L. Griffiths, J. G. J. Keene, J. H. Marshall, R. T. Platt, Michalina B. Pyzik, R. K. M. Sanders, J. K. Vowles, G. C. B. Winter. *In Biology* (*completing the Examination*) *:* A. Sander. *In Physics* (*completing the Examination*) *:* J. P. Fletcher, C. H. Thomas.

B.D.S.—*Second Examination :* A. H. Chivers, Ruth A. Yearn.

B.D.S.—*Third Examination :* J. S. Cooper. *In Dental Anatomy only :* D. C. Berry (with Distinction).

B.D.S.—*Final Examination. In Section II* (*Dental Surgery*) *:* G. K. G. Jennings. *In Section I only :* L. K. Walden.

L.D.S.—*Second Examination :* G. D. Everard, H. T. Jones, E. K. Joseph, G. D. Pamely, J. J. Tittle.

L.D.S.—*Third Examination :* J. P. B. Pengelly, J. T. Sanders. *In Dental Anatomy* (*completing the Examination*) *:* H. Sheffield. *In Dental Anatomy only :* Ruth Heller.

L.D.S.—*Final Examination. In Section II* (*Dental Surgery*) *:* R. J. Brownlee, T. C. Hughes, W. Watson. *In Section I only :* J. C. Fishpool.

D.P.H—.*Part II :* Joyce W. Brown, A. Donaldson, Clara J. Fraser, A. T. Hunt, S. Ludkin, D. G. MacNeill, Joan Williams.

D.P.M.—*Part I :* B. F. Martin, J. R. Stuart, L. B. Thomas. *In Anatomy and Physiology of the Nervous System* (*completing the Examination :* W. L. Jones. *In Psychology* (*completing the Examination*) *:* H. E. D. Flack, W. A. Heaton-Ward, H. A. Sutherland. *In Anatomy and Physiology of the Nervous System only :* A. Esterman, I. A. Macdonald. *In Psychology only :* M. W. Annear, I. M. Davies.

D.P.M.—*Part II :* G. M. Gibb, E. C. Kite, J. McGhie, J. F. D. Murphy.

HONOURS.

Congratulations are due to several of our old friends for honours conferred upon them.

Surgeon Vice-Admiral Henry St. Clair Colson, C.B., C.B.E., M.B., B.S., D.P.H., formerly S.R.A. at Barrow Gurney Hospital, who has been made K.C.B.

Colonel P. G. Stock, C.B., F.R.C.P., who has been made a C.M.G.

Dr. T. S. Hele, M.D., F.R.C.P., Master of Emmanuel College and Vice-Chancellor of Cambridge University, upon whom the King of Norway has bestowed the Order of St. Olaf, for his services to the Norwegians in Britain during the war.

Sir Lionel Whitby, C.V.O., M.D., F.R.C.P., Regius Professor of Physic at Cambridge University, who has been appointed Master of Downing College.

